# Boolean implication analysis unveils candidate universal relationships in microbiome data

**DOI:** 10.1186/s12859-020-03941-4

**Published:** 2021-02-05

**Authors:** Daniella Vo, Shayal Charisma Singh, Sara Safa, Debashis Sahoo

**Affiliations:** 1 Department of Bioinformatics and Systems Biology, University of California San Diego, La Jolla, CA 92093-083 USA; 2 Halıcıoğlu Data Science Institute, University of California San Diego, La Jolla, CA 92093-083 USA; 3 Department of Computer Science and Engineering, Jacob’s School of Engineering, University of California San Diego, La Jolla, CA 92093-083 USA; 4 Department of Pediatrics, University of California San Diego, 9500 Gilman Drive, MC 0730, Leichtag Building 132, La Jolla, CA 92093-083 USA; 5 Moores Cancer Center, University of California San Diego, La Jolla, CA 92093-083 USA

**Keywords:** Boolean analysis, Microbiome, Invariants, Systems biology, Microbe interactions

## Abstract

**Background:**

Microbiomes consist of bacteria, viruses, and other microorganisms, and are responsible for many different functions in both organisms and the environment. Past analyses of microbiomes focused on using correlation to determine linear relationships between microbes and diseases. Weak correlations due to nonlinearity between microbe pairs may cause researchers to overlook critical components of the data. With the abundance of available microbiome, we need a method that comprehensively studies microbiomes and how they are related to each other.

**Results:**

We collected publicly available datasets from human, environment, and animal samples to determine both symmetric and asymmetric Boolean implication relationships between a pair of microbes. We then found relationships that are potentially invariants, meaning they will hold in any microbe community. In other words, if we determine there is a relationship between two microbes, we expect the relationship to hold in almost all contexts. We discovered that around 330,000 pairs of microbes universally exhibit the same relationship in almost all the datasets we studied, thus making them good candidates for invariants. Our results also confirm known biological properties and seem promising in terms of disease diagnosis.

**Conclusions:**

Since the relationships are likely universal, we expect them to hold in clinical settings, as well as general populations. If these strong invariants are present in disease settings, it may provide insight into prognostic, predictive, or therapeutic properties of clinically relevant diseases. For example, our results indicate that there is a difference in the microbe distributions between patients who have or do not have IBD, eczema and psoriasis. These new analyses may improve disease diagnosis and drug development in terms of accuracy and efficiency.

## Background

In recent years, microbiome research has progressed rapidly, and there is an abundance of publicly available data. It is important to learn more about these microbes that are found in organisms and the environment, and the relationships between these microbes. There is also a growing interest to find the connection between microbiomes and diseases. Therefore, it is vital to efficiently analyze publicly available microbiome data focusing on microorganisms and their interactions with their host or environment.

### Current research and its limitations

Current methods of analysis fundamentally use Pearson’s correlation coefficient to determine relationships within a microbiome, such as in co-occurrence networks [1]. However, correlation only identifies relationships whose distribution is linear. This method may only work for data in which there is a linear relationship between the two variables bypassing data that is not fundamentally linear. Past studies may deduce weak correlations due to its nonlinearity thereby resulting in an incomplete analysis that overlooks critical components of the data. Additionally, there may be other types of relationships that cannot be identified through the standard methods of linear analysis currently in use. Correlation is also symmetric because corr(x, y) is same as corr(y, x). Therefore, asymmetric relationships are not captured well using correlation analysis.

Some forms of research used Boolean analysis to find links between oral microbiomes and HIV-associated periodontists [2] and to find a metabolic network of interactions in the gut microbiome [3]. However, these methods tend to analyze smaller datasets, which may have reproducibility issues. Additionally, these studies focus on specific areas such as the mouth and gut microbiomes.

### Boolean analysis is a more comprehensive approach

**Fig. 1:**
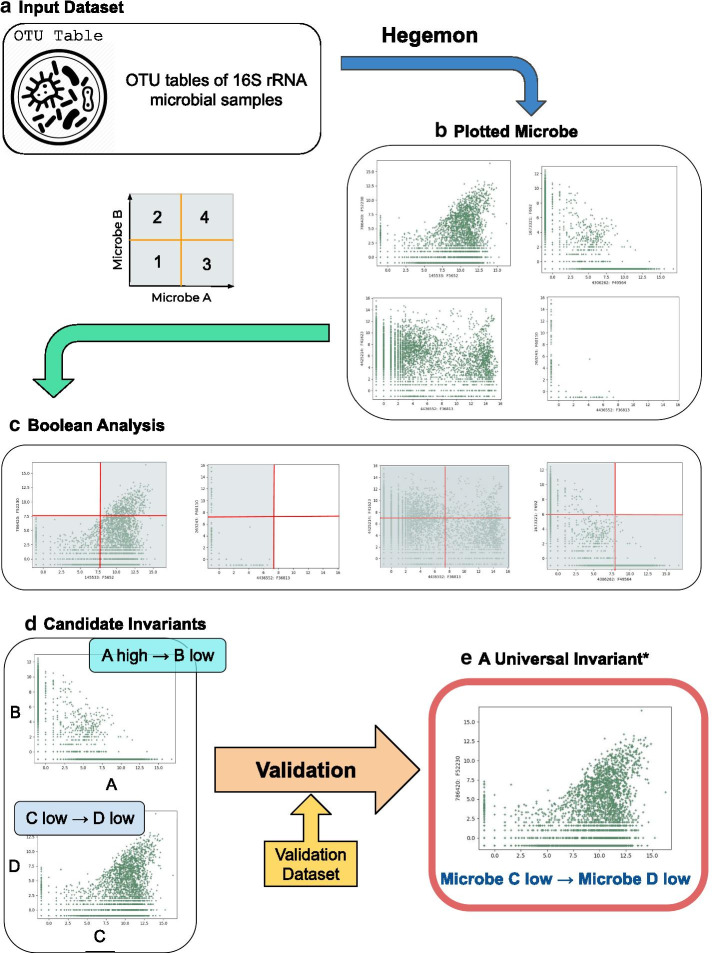
Study design. The overview of the research process: **a** OTU tables were collected from publicly available microbiome datasets. **b** Tables were uploaded to Hegemon and all possible microbe pairs were plotted (using 4 plots as examples—the number of total plots is larger). **c** Boolean analysis was performed on all the plots. **d** The plots that passed the BooleanNet statistics tests were marked as candidate invariants for further analysis and validation. **e** Any of the determined candidates that can be validated in other datasets, represents a likely universal invariant (a rule between two microbes that holds between them, any time the pair are present together in any environment). *Note that this is just an example. A universal invariant can be any of the 6 possible Boolean relationship

Instead of correlation-based analyses, we propose using Boolean analysis, a logical method to comprehensively study large amounts of microbiome data in order to determine dependencies between two variables. An overview of this methodology is described in Fig. 1. This method of Boolean implication analysis was successfully used to analyze relationships between genes to discover markers of blood stem cells [4], progenitors and a branch point in B-cell and T-cell differentiation [5], and has been applied in the study of colon [6, 7], bladder [8], and prostate cancer [9]. Since this method of Boolean analysis was previously used on gene expression data, we want to demonstrate the universality of this method by analyzing pairwise microbe relationships.

**Fig. 2:**
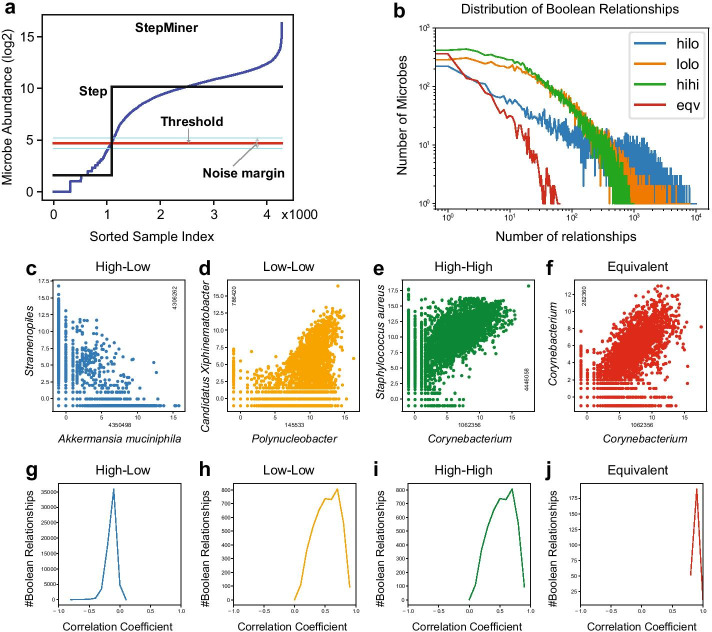
Boolean implication relationships represent diversity in microbiome data. All four types of Boolean relationships were found in our dataset. **a** Describes the StepMiner algorithm that creates thresholds for each microbe. Among all samples, the normalized counts (log_2_ scale) of a particular microbe are sorted, and a step function is fitted where the sharpest change between low microbe count and high microbe count takes place. The midpoint of the step position that minimizes the square error is chosen as the threshold (red line) for each respective microbe. A noise margin of ± 0.5 is considered around the threshold which is  ignored for Boolean analysis. **b** Depicts a log–log plot of the number of each type of relationship in the main dataset and the corresponding number of microbes that exhibit that specific relationship. Each of the four types of relationships found in our datasets are shown. Each point in the scatter plot corresponds to a sample, where the two axes represent the counts of each microbe. **c** high → low **d** low → low **e** high → high **f** equivalent. The remaining diagrams (**g**–**j**) show the correlation distributions according to the four Boolean implication relationships found in our datasets, with correlation on the x-axis and number of microbe pairs on the y-axis

Microbe normalized counts (in log_2_ scale) are first classified as either ‘low’ or ‘high’ using a threshold that is derived for each microbe species (Fig. 2a). An example of a Boolean implication rule is “if there is a high number of microbe A, then there will almost always be a low number of microbe B”, or A high → (implies) B low. There are six possible Boolean implication relationships: four asymmetric (A low → B low, A low → B high, A high → B low, and A high → B high) and two symmetric relationships (A equivalent B, and A opposite B). A low → B low is asymmetric because it is different from B low → A low, and the same applies to other asymmetric Boolean implication relationships. The convention used in this paper is to write Boolean implications with the microbe on the x-axis first; however, there exists a contrapositive relationship for each asymmetric Boolean implication, which is obtained by stating the microbe on the y-axis first and inverting low values to high values, and vice versa. For instance, A high → B low is identical to B high → A low.

Although our method of Boolean implication analysis has not been widely used in microbiology, a group of researchers attempted to use the method to perform analysis on microbiome data [10]. In their research, they analyzed environmental data from Visualization and Analysis of Microbial Population Structures (VAMPS) (http://vamps.mbl.edu/diversity/diversity.php) and focused specifically on marine microbes. Their research differs from ours in the aspect of diversity as we want to determine relationships that are present in a broader range of microbiomes, including humans, animals, and the environment. While this research demonstrates the impact Boolean implication analysis will have on microbiome analysis, we want to incorporate a larger and greater variety of samples. By having a wider array of samples, we want to find microbe relationships that not only exist in the environment, but also in humans and animals.

### Boolean methods have the potential to uncover universal invariants

Since Boolean implication analysis captures relationships that are often overlooked in the existing methods of analysis, we aim to uncover candidate invariants between pairs of microbes that are likely applicable to every microbe community. For example, if we find a recurring Boolean relationship between two microbe species in our large and diverse datasets, we expect this relationship to be a promising candidate invariant. This research includes more diverse datasets and a novel mathematical model compared to past studies, which helps produce stronger universal candidates.

The goal of this research is to comprehensively identify Boolean relationships that are likely universal. Through Boolean implication analysis, we will be able to determine candidate universal invariants within diverse microbe communities. Since these universal rules are expected to be robust, it can be translated to a clinical domain because fundamental rules should appear irrespective of diseases. These candidate universal invariants provide a basis in which scientists could use to determine how microbes are associated with diseases and make the process of identifying diseases easier because we expect these relationships to hold in the general population. Therapeutic use of microbes depends on their reproducibility in the general population, which makes our approach more suitable for discovering appropriate microbes.

## Results

**Fig. 3:**
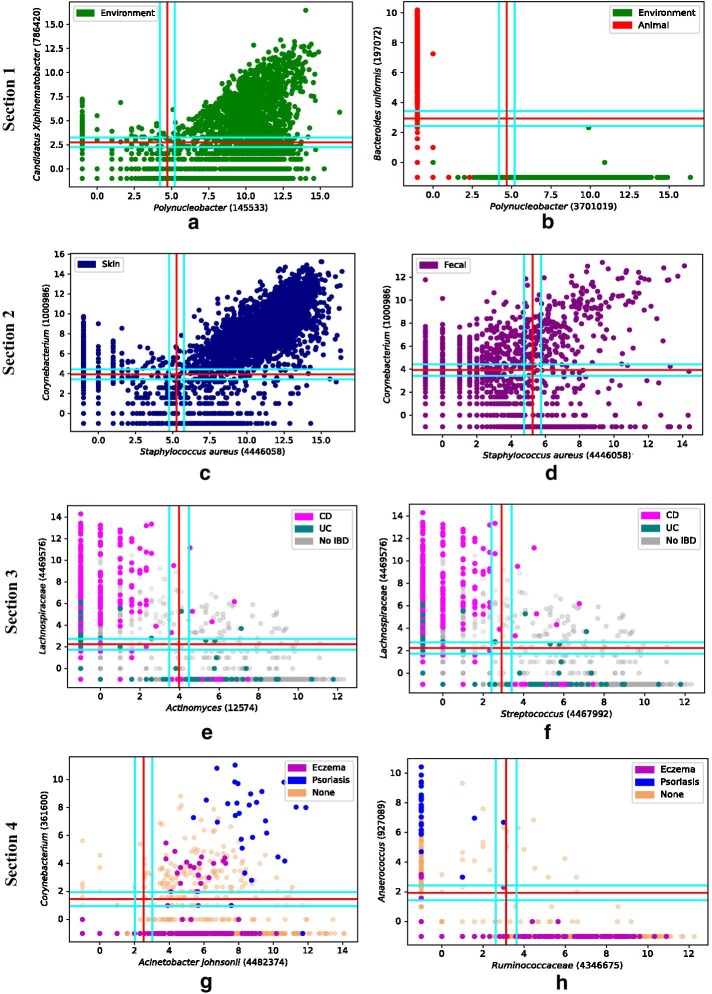
Boolean implications reveal strong patterns in diverse biological and environmental conditions. Analysis of scatter plots with various experimental conditions using metadata files that provided additional information about the samples. *Section 1*: green represents environmental samples (plants, water, soil, etc.) and red indicates animal samples (humans, animals). **a** *Polynucleobacter* (145533) low → *Candidatus Xiphinematobacter* (786420) low; this relationship is only present in environmental microbiomes due to the lack of red samples in the plot. **b** *Polynucleobacter* (3071019) high → *Bacteroides uniformis* (197072) low*;* this relationship suggests *Polynucleobacter* is mainly present in the environment, and *Bacteroides uniformis* is mainly present in animals. *Section 2*: **c** and **d** have the same microbes on the axes *Staphylococcus aureus* (446058) and *Corynebacterium* (1000986), but different regions of the body plotted: skin (dark blue) and feces (green). **c** shows the relationship *S. aureus* low → *Corynebacterium* low holds for the skin region. **d** shows the relationship using fecal samples, and there is no clear relationship that can be determined from this. *Section 3*: Pink represents Crohn’s Disease (CD), teal represents Ulcerative Colitis (UC), and light gray represents neither disease. **e** The relationship *Actinomyces* (12574) high → *Lachnospiraceae* (4469576) low is shown, with higher counts of *Lachnospiraceae* in CD, and higher counts of *Actinomyces* in UC. **f** shows the relationship *Streptococcus* (4467992) high → *Lachnospiraceae* (4469576) low, with higher counts of *Lachnospiraceae* in CD, while higher counts of Streptococcus in UC. *Section 4*: Magenta represents eczema, blue represents psoriasis, and beige represents neither skin condition. **g** The relationship *Acinetobacter johnsonii* (4482374) low → *Corynebacterium* (361600) low is shown. Patients with psoriasis tend to have higher counts of* Corynebacterium* than patients with eczema. **h** The relationship *Ruminococcaceae* (4346675) high → *Anaerococcus* (927089) low is shown. Patients with psoriasis tend to have higher counts of *Anaerococcus*, while patients with eczema tend to have higher counts of *Ruminococcaceae*

Many universal Boolean relationships were uncovered using the proposed method of Boolean implication analysis. These pooled datasets are comprised of a variety of environmental, animal, and human samples. A main dataset was used to perform Boolean analysis for approximately 365 million microbe pairs and we discovered about 27 million relationships (Fig. 2b) with a high statistical significance (false discovery rate (FDR) of 2.3 × 10^–4^). We also used three pooled independent datasets to validate these relationships (with FDRs of 3.3 × 10^–4^, 4.4 × 10^–4^ and 2.1 × 10^–4^ respectively), and found approximately 330,000 relationships that were consistent throughout all four datasets. We present some relationships in Figs. 2 and 3 that have a superior BooleanNet statistics (S, p) with higher independence statistic (S), lower error rate (p) and an underlying biological relevance. Figure 2 is mainly used to present the possible types of Boolean relationships found in microbiomes while Fig. 3 displays some of the biological properties associated with certain microbes.

### Boolean implication relationships are conserved across environments and species

The high → low Boolean implication shows a high count of *Akkermansia muciniphila* (Operational Taxonomic Unit (OTU) ID 4306262) implying a low count of Stramenopiles (OTU ID 4350498) (Fig. 2c). The contrapositive relationship also holds (Stramenopiles high → *A. muciniphila* low)*. A. muciniphila* is a human gut bacterium linked to preventing obesity, diabetes, and inflammation [11] and Stramenopiles is found in aquatic environments, mostly made up of algae [12]. Since these two microbes are rarely found in similar environments, it makes sense that when one microbe’s frequency is high, the other is low. This logic is consistent with the high → low Boolean relationship found.

The graph in Fig. 2d displays a strong low → low relationship, showing that when *Polynucleobacter* (OTU ID 145533) is low, *Candidatus Xiphinematobacter* (OTU ID 786420) is also low. This relationship is confirmed in other studies which found *Polynucleobacter* makes up a large portion of freshwater bacterioplankton [13] and *Candidatus Xiphinematobacter* is a known nutrient supplier to nematodes, which are abundant in freshwater environments [14]. It is presumable that a low count of *Polynucleobacter* indicates an environment that does not contain freshwater; therefore, it is unlikely that the count of *Candidatus Xiphinematobacter* is high, further confirming this low → low Boolean implication.

### Boolean relationships confirm some known biological properties

A strong high → high relationship was found is between *Corynebacterium* (OTU ID 1062356) and *Staphylococcus aureus* (OTU ID 4446058) (Fig. 2e). *Corynebacterium* and *S. aureus* species both reside in the nose trail and skin microbiota of humans. *S. aureus* can be pathogenic and can cause infections. Studies have shown that *Corynebacterium* spp. and *S. aureus* reside together, indicating that they are positively correlated [15]. In addition to being positively correlated, the *Corynebacterium* high → *S. aureus* high relationship reveals that it is also possible to have a low count of *Corynebacterium* and a high count of *S. aureus.*

An example of a symmetric relationship is shown in Fig. 2f, where *Corynebacterium* (OTU ID 1062356) is equivalent to *Corynebacterium* (OTU ID 282360). *Corynebacterium* are a family of Gram-positive bacteria with a large number of known species which are of interest in the medical field [16]. However, the specific species of these *Corynebacterium* are not stated in the GreenGenes database. Further analysis could determine the specific *Corynebacterium* species which would allow us to confirm this symmetric relationship. Although the specific species in the database are unknown, there is a symmetric relationship between the two species such that as the count of one species increases so does the other.

### Microbes yield different Boolean implications in environmental versus animal samples

In the next two examples in Section 1 of Fig. 3, green represents environmental samples and red represents both human and animal samples. Figure 3a shows the relationship *Polynucleobacter* (OTU ID 145533) low → *Candidatus Xiphinematobacter* (OTU ID 786420) low. The abundance of green samples suggests that this relationship is mainly present in environmental microbiomes and is not typically present in animal microbiomes. As stated previously, *Polynucleobacter* makes up a large portion of freshwater bacterioplankton [13], while *Candidatus Xiphinematobacter* tends to be found in soil samples [17], meaning they are both environmental microbes and are not typical microbes found in humans or animals.

Figure 3b presents the relationship *Polynucleobacter* (OTU ID 3071019) high → *Bacteroides uniformis* (OTU ID 197072) low. This Boolean relationship suggests that *Polynucleobacter* is mainly present in the environmental microbiome while *Bacteroides uniformis* mostly exist in the animal microbiome. Previous studies have shown that *Bacteroides uniformis* is one of the main bacterial species of the human gut microbiome [18].

### Different body regions affect the presence of microbes relationships

In Section 2 of Fig. 3, both plots show the relationship between *Staphylococcus aureus* (OTU ID 446058) and *Corynebacterium* (OTU ID 1000986) but in different regions of the human body. While Fig. 3c suggests that *S. aureus* and *Corynebacterium* have a low → low relationship in the skin region, based on Fig. 3d, there is no specific Boolean relationship between these two microbes when they are present in human feces. Specific regions may have differing relationships due to the proclivity microbes have towards one region versus another.

### Boolean implications using disease-specific microbes is promising in potential diagnosis

Inflammatory bowel disease (IBD) is a gastrointestinal disorder that is currently difficult to treat, but treatments using the gut microbiome have been proposed [19]. The relationship *Actinomyces* (OTU ID 12564) high → *Lachnospiraceae* (OTU ID 4469576) low (Fig. 3e) specifically highlights samples from patients that either have Crohn’s Disease (CD), Ulcerative Colitis (UC), or neither (No IBD). There tends to be a higher proportion of *Lachnospiraceae* in patients with CD than UC, and a higher proportion of *Actinomyces* in patients of UC versus CD. The relationship *Streptococcus* (OTU ID 4467992) high → *Lachnospiraceae* (OTU ID 4469576) low (Fig. 3f) also highlights differences between samples of IBD patients. A similar trend of a higher proportion of *Lachnospiraceae* in CD patients and a higher proportion of *Streptococcus* in UC patients appears with these microbes.

Certain microbes seem to be related to skin conditions, such as eczema and psoriasis. The relationship *Acinetobacter johnsonii* (OTU ID 4482374) low → *Corynebacterium* (OTU ID 361600) low shows how patients with psoriasis tend to have higher counts of both *A. johnsonii* and *Corynebacterium* than patients with eczema and patients with neither skin condition. In looking at another relationship, *Ruminococcaceae* (OTU ID 4346675) high → *Anaerococcus* (OTU ID 927089) low, it is clear that patients with psoriasis have higher counts of *Anaerococcus,* while patients with eczema have higher counts of *Ruminococcaceae*, with both having minimal amounts of the other microbes. Our method of Boolean implication analysis attempts to provide a mathematical model of identifying candidate universal invariants. This will enable the determined microbiome properties to apply in almost all states, and hopefully provide treatments for such diseases to be universally successful.

## Discussion

Boolean implication analysis can be used to comprehensively determine microbial relationships, which can then be used to build abstract versions of biological systems. Understanding and simulating biological systems has always been the goal of researchers, but current analysis has not met that objective with simplified symmetric analysis and smaller volume of datasets. Relationships between microbes and diseases have always been evident, so our research intends to build the foundation of the biological system.

### Correlation versus Boolean implication analysis

In a correlation-based analysis, we found that most of the microbe pairs that exhibited an asymmetric Boolean relationship had a weak correlation. As expected, equivalent Boolean relationships (which are symmetric relationships) typically contain highly correlated microbes. Figure 2g–j depicts the distribution of correlation values for each of the four Boolean relationships found in our dataset. It was rare to find asymmetric microbe pairs that yielded a strong correlation which further highlights the shortcomings of analyses based on correlations. This reinforces the observation that studying these relationships may result in a more comprehensive and complete analysis.

Our results reveal that Boolean analysis is a promising method for analyzing different microbiomes. After analyzing more than 400 diverse datasets consisting of over 100,000 samples, we uncovered candidate invariants that held in all our datasets, which is consistent with our hypothesis. However, only four of the six Boolean implication relationships were found in the datasets: low → low, high → low, high → high, and equivalent. The other two relationships, opposite and low → high, did not appear in the datasets because in these two relationships, the low, low quadrant is sparse. A sparse low, low quadrant means both microbes cannot exist in low amounts at the same time. Due to the diverse nature of microbes, we believe that it should be rare for the low, low quadrant to be sparse which justifies the lack of low → high and opposite relationships in our results.

### Boolean analysis unveils differences in microbe interactions due to environment, body site, and disease

Environmentally distinct microbes exhibit relationships such as *A. muciniphila* high → Stramenopiles low because *A. muciniphila* is present in the human gut [11] while Stramenopiles is present in aquatic environments [12]. Boolean analysis also highlighted the dichotomies in different body sites, as some relationships are present in certain regions of the body, but not in others. The* S. aureus* (OTU 4446058) low → *Corynebacterium* (1000986) low relationship observed in skin samples is supported by biological research [15] but is not present in fecal samples. Boolean analysis allows us to see where a clinically relevant relationship might occur in the body. The relationships *A. johnsonii* low → *Corynebacterium* low and *Ruminococcaceae* high → *Anaerococcus* low reveal that samples from eczema and psoriasis were clustered in certain regions of the graph. Although there has been research to detect microbial diversity on the skin, experts still cannot agree on a universal method of using microbes to diagnose psoriasis in patients [20]. Using the clustered regions from eczema and psoriasis-specific relationships, scientists would be able to define healthy and diseased ranges for microbial frequencies which is promising for disease diagnosis.

Scientists can develop therapeutics targeting diseases like IBD using relationships such as *Actinomyces* high → *Lachnospiraceae* low and *Streptococcus* high → *Lachnospiraceae* low. Numerous studies show that there is a decreased amount of Clostridiales (*Lachnospiraceae* is in the class Clostridiales) in patients with Irritable Bowel Syndrome (IBS) [19, 21–23], but there is limited information about IBD. Although there have been studies showing a connection between *Actinomyces* and infections [24], studies have not compared the amount of *Actinomyces* to IBD. However, our results suggest that Clostridiales and *Actinomyces* are connected to UC and CD. There is no previous research suggesting there is a connection between *Streptococcus* and IBD, indicating a need for more research to determine if this relationship has any disease-identifying properties. Relationships like these might have only been discovered using methods that consider asymmetry like Boolean implication analysis which might explain why no studies can confirm such relationships.

### Limitations of Boolean implication analysis

One of the limitations of Boolean analysis is that the data focuses on the stronger relationships making the analysis less noisy and weaker relationships are lost in the process. Further analysis might prove whether these weaker relationships have significance, but this method focuses on the stronger relationships. A second limitation is that we only analyzed datasets downloaded from Qiita that were processed using the GreenGenes database [25]. However, the latest version of GreenGenes was published in 2013, which may not include the most up to date information involving microbiome taxonomy. Additionally, since we are not limiting the scope to microbiomes found in a specific region, but focusing on microbes found universally in humans, animals, and the environment, we could be excluding compelling relationships that are only found in specific regions. Future studies using this method of Boolean analysis can be done to focus on these specific regions to provide better insight into particular microbiomes, such as the gut microbiome.

## Conclusions

The lack of comprehensive analysis of microbiome data created a need for more extensive approaches. Boolean implication analysis presents a solution that incorporates both symmetric and asymmetric relationships. Our results show that some biological properties were confirmed by Boolean analysis. For example, it is proven that the *Corynebacterium* and *S. aureus* species reside together and are positively correlated, which is consistent with the high → high relationship found. Our results also show that different microbiomes affect the presence of microbe relationships on a broader scope, such as environmental versus animal samples, and on a smaller scope, such as various body sites. Boolean implication analysis is promising in terms of potential disease diagnosis including IBD. We found that higher frequencies of certain microbes seem to be associated with either CD or UC.

Each implication is believed to be a universal candidate because it holds in all the datasets we analyzed. These microbe relationships can be validated in the lab by generating and sequencing additional samples to confirm these relationships. Future work also includes building a Boolean implication network to further analyze how microbe implications are connected to each other. A Boolean implication network with the candidate microbe invariants may help in developing better models for biological systems. Our research also helps determine strong properties of biological systems and future research on this topic provide novel directions in understanding how these systems work. Invariants help formulate new theories that may provide more effective diagnostic and therapeutic applications.

## Methods

### Data collection

We extracted pre-processed OTU tables along with the corresponding metadata from Qiita [26], a microbiome database and study management platform. Qiita uses third party plugins including QIIME (Quantitative Insights Into Microbial Ecology) (http://qiime.org/) or QIIME 2 (https://qiime2.org/) to process microbial 16S rRNA sequences of each study, which are contributed by users on this platform. Qiita classifies the microbes using the GreenGenes database and generates OTU tables. OTU tables display the counts of all the microbe species present in every sample. Each study and the corresponding raw count data comes from different individuals and institutions, which makes our analysis comprehensive. The metadata includes information about the samples, such as location and sample identification. For easier analysis of the collected data, we separated the downloaded studies from QIITA and pooled them into four independent datasets. We performed our main analysis on one of the pooled datasets and used the other three datasets for validation. In Additional file 1: Table S1, we specify all the studies used and which pooled dataset it belongs to (main, validation datasets 1, 2 or 3). Data from the main dataset is presented in the figures unless explicitly stated otherwise. The data from the OTU tables and metadata are transformed using a log_2_ scale and were subsequently uploaded onto a web-based tool for analyzing big data called Hegemon [7, 8, 27–29].

### StepMiner algorithm

To classify a relationship, thresholds are first determined for each microbe using the StepMiner algorithm. The StepMiner algorithm [30] is a tool that helps identify stepwise transitions (either step-up or step-down transitions) calculated using sum-of-square errors. After the data is normalized, steps are defined as the sharpest change between low microbe count and high microbe count. In order to fit a step function, the StepMiner algorithm computes the average of the values on both sides of the step for all possible step positions. The midpoint of the step position that minimizes the square error is chosen as the threshold for each respective microbe. The step is placed at the largest jump from low values to high values and sets the threshold at the point where the step crosses the original data. The microbe counts are normalized and transformed into log_2_ scale before Boolean analysis. Microbe counts (in log_2_ scale) are further classified as either ‘high’, ‘low’, or ‘intermediate’. If *t* is the microbe count threshold, levels above *t* + 0.5 are ‘high’, levels below *t* – 0.5 are ‘low’, and levels between *t* – 0.5 and *t* + 0.5 are ‘intermediate’. Points in the intermediate region are ignored because these points might appear on either side of the threshold due to noise.

### Boolean implication analysis

There are six possible Boolean implications: symmetric (opposite and equivalent) or asymmetric (low → low, low → high, high → low, high → high). The asymmetric relationships are determined by checking if one of the four quadrants in the scatter plot is significantly sparse compared with other quadrants. If A low → B low and A high → B high are both sparsely populated, then A is equivalent to B. If A high → B low and A low → B high are both sparsely populated, then A is opposite to B. The BooleanNet statistic tests [27] determine whether there is a Boolean relationship between A and B. Consider the relationship A low → B high. First, test if the microbe counts in the sparse quadrant are significantly less than the expected counts in an independence model. Let a_00_, a_01_, a_10_, and a_11_ represent the quadrants in which the microbe counts of A and B are low and low, low and high, high and low, and high and high, respectively.

$$total={a}_{00}+{a}_{01}+{a}_{10}+{a}_{11}$$

$$\mathrm{number of A low counts }= n{A}_{low}=({a}_{00}+{a}_{01})$$

$$\mathrm{number of B low counts }= n{B}_{low}=({a}_{00}+{a}_{10})$$

$$expected=\left(\frac{n{A}_{low}}{total}\times \frac{n{B}_{low}}{total}\right)\times total=\left({a}_{00}+{a}_{01}\right)\times \frac{\left({a}_{00}+{a}_{10}\right)}{total}$$

$$observed={a}_{00}$$

$$S\; statistic=\frac{expected-observed}{\sqrt{expected}}$$

Second, the observed values in the sparse quadrant are not ideal for Boolean implication formula. They are assumed to be erroneous points for the purpose of analysis only as was described previously in the context of gene expression analyses [27]. However, these points may or may not be erroneous from a real biological point of view. We wanted to discover the general trends of Boolean implication relationships with such a strong assumption. A maximum likelihood estimate of this error rate is then computed:

$$error\;rate=\frac{1}{2}(\frac{{a}_{00}}{{a}_{00}+{a}_{01}}+\frac{{a}_{00}}{{a}_{00}+{a}_{10}})$$

If both tests succeed, the low-low quadrant is considered sparse, so the implication A low → B high is true. An implication is considered significant if the S statistic is greater than 3 and the error rate is less than 0.1. Microbe relationships that pass both of these tests are now considered candidate invariants.

The OTU tables are then uploaded onto Hegemon to visualize the Boolean relationships on scatter plots for comparing two microbes against each other. In each graph, one microbe species’ counts (using OTU ID A) is plotted on the x-axis, and another microbe species’ counts (OTU ID B) is plotted on the y-axis. Each data point represents a sample and the counts are plotted on a log–log scale. Using the graphs constructed on Hegemon, we can visually confirm if a Boolean implication relationship, determined using the BooleanNet statistics, is present.

### Metadata analysis

We used a Hegemon function that calculates the differential analysis for specific factors in the metadata using t-tests in R software framework (R version 3.4.4—2018–03-15). We then selected the OTU IDs that had higher mean differential values and higher –log_10_(p) values. After the list of potential IDs were generated, we visually confirmed on Hegemon whether certain factors such as environment versus animal or body site affects the microbe counts.

### Computation of false discovery rate

To evaluate the significance of the Boolean implication relationships found, we computed the FDR for each of the pooled datasets by randomly permuting the counts for each microbe independently 10 times and determining the Boolean relationships for this randomized dataset using the method described above. The FDR is the ratio of the average number of Boolean relationships in the randomized dataset to the original dataset.

### Correlation analysis

Correlation analysis was conducted on a subset of 500 microbes from the main dataset. The Pearson correlation coefficients and Boolean relationships for each pair of microbes were calculated to compare these two methods of analysis. The package “pearsonr” from the python library “scipy.stats” was used to perform the calculations.

## Supplementary Information

**Additional file 1**. List of QIITA study accession numbers used in the four different pooled datasets (main, validation datasets 1, 2 or 3).

## Data Availability

All datasets are publicly available from https://qiita.ucsd.edu, under the Study, and View Studies tab. We selected a subset of all the available studies. The specific list of studies used for this research is included as an additional file.

## Abbreviations

CD: Crohn’s disease
FDR: False discovery rate
IBD: Inflammatory bowel disease
IBS: Irritable bowel syndrome
QIIME: Quantitative insights into microbial ecology
OTU: Operational taxonomic units
UC: Ulcerative colitis
VAMPS: Visualization and analysis of microbial population structures

## References

[1] Bauer E, Thiele I (2018). From network analysis to functional metabolic modeling of the human gut microbiota. mSystems..

[2] Noguera-Julian M, Guillén Y, Peterson J, Reznik D, Harris EV, Joseph SJ (2017). Oral microbiome in HIV-associated periodontitis. Medicine.

[3] Steinway SN, Biggs MB, Loughran TP, Papin JA, Albert R (2015). Inference of network dynamics and metabolic interactions in the gut microbiome. PLoS Comput Biol.

[4] Chen JY, Miyanishi M, Wang SK, Yamazaki S, Sinha R, Kao KS (2016). Hoxb5 marks long-term haematopoietic stem cells and reveals a homogenous perivascular niche. Nature.

[5] Inlay MA, Bhattacharya D, Sahoo D, Serwold T, Seita J, Karsunky H (2009). Ly6d marks the earliest stage of B-cell specification and identifies the branchpoint between B-cell and T-cell development. Genes Dev.

[6] Dalerba P, Sahoo D, Clarke MF (2016). CDX2 as a prognostic biomarker in colon cancer. N Engl J Med.

[7] Dalerba P, Sahoo D, Paik S, Guo X, Yothers G, Song N (2016). CDX2 as a prognostic biomarker in stage II and stage III colon cancer. N Engl J Med.

[8] Volkmer JP, Sahoo D, Chin RK, Ho PL, Tang C, Kurtova AV (2012). Three differentiation states risk-stratify bladder cancer into distinct subtypes. Proc Natl Acad Sci U S A.

[9] Sahoo D, Wei W, Auman H, Hurtado-Coll A, Carroll PR, Fazli L (2018). Boolean analysis identifies CD38 as a biomarker of aggressive localized prostate cancer. Oncotarget.

[10] Zhu C, Jiang R, Chen T (2014). Constructing a Boolean implication network to study the interactions between environmental factors and OTUs. Quantitative Biology.

[11] Depommier C, Everard A, Druart C, Plovier H, Van Hul M, Vieira-Silva S (2019). Supplementation with *Akkermansia muciniphila* in overweight and obese human volunteers: a proof-of-concept exploratory study. Nat Med.

[12] Derelle R, López-García P, Timpano H, Moreira D (2016). A phylogenomic framework to study the diversity and evolution of Stramenopiles (=Heterokonts). Mol Biol Evol.

[13] Hahn MW, Jezberová J, Koll U, Saueressig-Beck T, Schmidt J (2016). Complete ecological isolation and cryptic diversity in Polynucleobacter bacteria not resolved by 16S rRNA gene sequences. ISME J.

[14] Brown AM, Howe DK, Wasala SK, Peetz AB, Zasada IA, Denver DR (2015). Comparative genomics of a plant-parasitic nematode endosymbiont suggest a role in nutritional symbiosis. Genome Biol Evol.

[15] Ramsey MM, Freire MO, Gabrilska RA, Rumbaugh KP, Lemon KP (2016). *Staphylococcus aureus* shifts toward commensalism in response to corynebacterium species. Front Microbiol.

[16] Liebl W (2005). Corynebacterium taxonomy. Handbook of Corynebacterium glutamicum.

[17] Lazarova S, Peneva V, Kumari S (2016). Morphological and molecular characterisation, and phylogenetic position of X. browni sp. n., X. penevi sp. n. and two known species of *Xiphinema americanum*-group (Nematoda, Longidoridae). Zookeys.

[18] Renouf M, Hendrich S (2011). Bacteroides uniformis is a putative bacterial species associated with the degradation of the isoflavone genistein in human feces. J Nutr.

[19] Pittayanon R, Lau JT, Yuan Y, Leontiadis GI, Tse F, Surette M (2019). Gut microbiota in patients with irritable bowel syndrome—a systematic review. Gastroenterology.

[20] Langan EA, Griffiths CEM, Solbach W, Knobloch JK, Zillikens D, Thaçi D (2018). The role of the microbiome in psoriasis: moving from disease description to treatment selection?. Br J Dermatol.

[21] Jalanka-Tuovinen J, Salojärvi J, Salonen A, Immonen O, Garsed K, Kelly FM (2014). Faecal microbiota composition and host-microbe cross-talk following gastroenteritis and in postinfectious irritable bowel syndrome. Gut.

[22] Carroll IM, Ringel-Kulka T, Siddle JP, Ringel Y (2012). Alterations in composition and diversity of the intestinal microbiota in patients with diarrhea-predominant irritable bowel syndrome. Neurogastroenterol Motil..

[23] Li G, Yang M, Jin Y, Li Y, Qian W, Xiong H (2018). Involvement of shared mucosal-associated microbiota in the duodenum and rectum in diarrhea-predominant irritable bowel syndrome. J Gastroenterol Hepatol.

[24] Könönen E, Wade WG (2015). Actinomyces and related organisms in human infections. Clin Microbiol Rev.

[25] DeSantis TZ, Hugenholtz P, Larsen N, Rojas M, Brodie EL, Keller K (2006). Greengenes, a chimera-checked 16S rRNA gene database and workbench compatible with ARB. Appl Environ Microbiol.

[26] Gonzalez A, Navas-Molina JA, Kosciolek T, McDonald D, Vázquez-Baeza Y, Ackermann G (2018). Qiita: rapid, web-enabled microbiome meta-analysis. Nat Methods.

[27] Sahoo D, Dill DL, Gentles AJ, Tibshirani R, Plevritis SK (2008). Boolean implication networks derived from large scale, whole genome microarray datasets. Genome Biol.

[28] Sahoo D, Seita J, Bhattacharya D, Inlay MA, Weissman IL, Plevritis SK (2010). MiDReG: a method of mining developmentally regulated genes using Boolean implications. Proc Natl Acad Sci U S A.

[29] Dalerba P, Kalisky T, Sahoo D, Rajendran PS, Rothenberg ME, Leyrat AA (2011). Single-cell dissection of transcriptional heterogeneity in human colon tumors. Nat Biotechnol.

[30] Sahoo D, Dill DL, Tibshirani R, Plevritis SK (2007). Extracting binary signals from microarray time-course data. Nucleic Acids Res.

